# Marital satisfaction and phubbing: the roles of depressive symptoms and subjective social status

**DOI:** 10.3389/fpsyt.2026.1868381

**Published:** 2026-09-15

**Authors:** Xiwu Zhang, Ru Zhou, Wei Zhang, Wenhua Zhang

**Affiliations:** 1 Department of Pharmacy, Shandong Provincial Hospital Affiliated to Shandong First Medical University, Jinan, Shandong, China; 2 School of Psychology, Northeast Normal University, Changchun, Jilin, China; 3 Psychology Research and Training Center, Dalian Education University, Dalian, Liaoning, China; 4 Department of Social Medicine and Health Management, School of Public Health, Cheeloo College of Medicine, Shandong University, Jinan, Shandong, China; 5 Department of Obstetrics and Gynecology, Qilu Hospital of Shandong University, Jinan, Shandong, China; 6 Nursing Department, Qilu Hospital of Shandong University, Jinan, Shandong, China; 7 Nursing Theory and Practice Innovation Research Center, Shandong University, Jinan, Shandong, China

**Keywords:** depressive symptoms, marital satisfaction, moderated mediation model, phubbing, subjective social status

## Abstract

**Background:**

Previous studies have mainly examined the associations between partner phubbing and relationship outcomes among phubbees, whereas less is known about how marital relationship quality is associated with phubbing among phubbers themselves. This study aims to examine the relationship between marital satisfaction and phubbing among Chinese married adults, and examine the mediating role of depressive symptoms, as well as the moderating role of subjective social status (SSS) in this association.

**Methods:**

A cross-sectional survey was conducted with 565 married adults in China (31.85 ± 4.85 years; 67.3% female). Participants completed self-report measures of marital satisfaction, depressive symptoms, phubbing, and SSS. Moderated mediation analyses were conducted to examine the indirect role of depressive symptoms and the conditional indirect associations across levels of SSS.

**Results:**

Depressive symptoms significantly mediated the association between marital satisfaction and phubbing. SSS moderated both the direct path from marital satisfaction to phubbing and the second stage of the mediation model from depressive symptoms to phubbing. Simple slope analyses revealed that marital satisfaction was negatively associated with phubbing only among individuals with a high SSS (β*_simple_* = -0.42, *t* = -4.16, *p* < 0.001). Furthermore, depressive symptoms were positively associated with phubbing among adults with low SSS (β*_simple_* = 0.62, *t* = 6.99, *p* < 0.001), while this association was not significant among those with high SSS (M + 1SD) (β*_simple_* = 0.11, *t* = 1.13, *p* = 0.26).

**Conclusion:**

Depressive symptoms accounted for part of the indirect association between lower marital satisfaction and greater phubbing among Chinese married adults, and this process varies as a function of SSS. Prevention and intervention efforts targeting phubbing in marital contexts may benefit from considering both depressive symptoms and individuals’ perceived social status.

## Background

Many individuals give a high priority to regular mobile phone use due to its multi-functional nature and relevance to daily living (1). With 5G capabilities gradually becoming standard across mobile phones, higher quality services are now widely accessible. People of all ages are increasingly spending time interacting with mobile devices (2, 3). While smartphones offer significant convenience to modern life, the phenomenon of phubbing, a portmanteau of “phone” and “snubbing”, has emerged as an inevitable behavioral pattern (4). Phubbing describes the act of checking the phone during face-to-face conversation rather than paying attention to interpersonal communication (5). Although phubbing has been discussed alongside constructs such as problematic smartphone, Internet, social media, and gaming use (6), it remains conceptually distinct from these and is not currently established as a clinically recognized addictive or diagnostic entity. Phubbing has been characterized as a disrespectful behavior that may share features with problematic technology use (6, 7), which undermines the quality of interpersonal relationships and face-to-face communication (8, 9). Given these negative consequences, identifying the factors and correlates associated with phubbing is crucial for developing effective prevention and intervention strategies.

According to the bioecological theory of human development, the family represents a primary environmental microsystem that interconnects with other ecological systems to influence the development of family members (10). Marital satisfaction is best understood as “a psychological state regulated by mechanisms that monitor the benefits and costs of marriage to a particular person” (11) and serves as an important parameter to evaluate the emotions of married people (12). Previous empirical research has demonstrated that lower marital satisfaction is associated with various negative outcomes, including addictive gaming behavior (13). From the perspective of interpersonal theory, individuals with unmet marital relationship needs may seek compensation through cyberspace, where they can establish cyber-relationships that provide a sense of belonging (14, 15). Additionally, excessive smartphone use or phubbing may serve as a coping mechanism to reduce negative affect caused by low marital satisfaction (16). Chen et al. (17) provided prospective evidence for this directionality in a two-wave longitudinal study among Chinese married couples, demonstrating that relationship quality negatively predicted phubbing behavior for both husbands and wives. This finding suggests that marital dissatisfaction may function as an antecedent of phubbing rather than merely a consequence of it. It is important to distinguish between the consequences of being phubbed (phubbee perspective) and the antecedents of phubbing itself (phubber perspective). Existing studies have largely examined how partner phubbing undermines relationship satisfaction among those who are phubbed (18, 19), or have examined related family processes, such as the association between parental marital conflict and children’s Internet addiction (20). However, little is known about how marital dissatisfaction may be associated with phubbing among married adults themselves, and the factors and processes underlying this association remain largely underexplored. Given that marital dissatisfaction may generate negative emotional experiences, depressive symptoms may be an important explanatory variable linking marital satisfaction to phubbing.

Depressive symptoms are characterized as a psychological state involving persistent negative mood, reduced interest in activities, and diminished sense of well-being. A meta-analysis including 26 cross-sectional studies confirmed a significant relationship between marital satisfaction and depression (21). Longitudinal studies also revealed that marital satisfaction predicted depressive symptoms at one to seven years later (22–26). According to the marital discord model of depression (MDMD), marital dissatisfaction may increase the risk of depressive symptoms by undermining spousal support, dependability and intimacy, while heightening stress and hostility between partners (27, 28). Furthermore, depressive symptoms have also been significantly associated with increased phubbing (29, 30), suggesting that individuals experiencing elevated depressive symptoms are more inclined to engage in phubbing. According to the cognitive-behavioral model of pathological Internet use, depressive symptoms may function as a distal predisposing factor for pathological Internet use and behavioral addiction (31). To the extent that phubbing involves excessive smartphone engagement that displaces face-to-face interaction, this model offers a useful lens for understanding depressive symptoms as a potential distal factor associated with phubbing. Our findings support depressive symptoms as a plausible mediator of the association between marital satisfaction and phubbing.

Not all individuals with low marital satisfaction develop depressive symptoms or engage in phubbing, suggesting the presence of protective factors that could buffer against adverse outcomes. In addition to objective status, individuals also form a subjective sense of their standing relative to others, referred to as subjective social status (SSS). SSS is conceptually distinct from objective socioeconomic status (SES), such as income, occupation or education. Whereas SES reflects verifiable economic and educational resources, SSS captures individuals’ own perception of their position in the social hierarchy (32) and may not correspond directly to their objective SES. Despite this conceptual distinction, SSS has been shown to predict health outcomes, including mental, physical, and behavioral health, beyond what objective SES accounts for (33–35). Prior studies have demonstrated that SES moderated the relationship between marital variables and outcomes. For instance, the association of marital quality and life satisfaction is moderated by perceived SES (36), marital conflict is more detrimental to health among those with lower SES (37), and the relationship between marital status and suicide risk varies by SES (38). Lower SSS is associated with chronic stress, reduced feelings of security, and limited economic, social, and cultural resources (39–41). Cumulative risk models indicate that exposure to multiple risk factors (such as low marital satisfaction combined with lower SSS) produces worse outcomes (42). Despite this evidence, SSS has rarely been examined as a moderating factor in the associations between marital satisfaction and both depressive symptoms and phubbing.

Although phubbing has been studied across various cultural contexts, its manifestation and underlying patterns may differ substantially between Western and Eastern populations. In Western countries such as Germany, research indicates that younger adults check their smartphones more than 10 times per day (43, 44), while problematic Internet use and smartphone addiction rates remain relatively lower compared to East Asian countries (45). China has one of the largest smartphone user populations in the world, with studies consistently reporting higher rates of Internet addiction and problematic smartphone use (46) among Chinese adults and adolescents compared to their counterparts from Singapore and the United States (47). These differences may be partly attributable to cultural factors. Chinese society, shaped by Confucian values, places strong emphasis on collectivism, filial piety, and harmonious interpersonal relationships, which may render Chinese adults particularly sensitive to marital discord and relational dissatisfaction. Within this cultural context, phubbing may carry distinct interpersonal meaning, as it represents a withdrawal from face-to-face interaction that directly conflicts with culturally valued norms of relational attentiveness.

Furthermore, prior research has demonstrated that marital satisfaction tends to decline after the birth of a child, partly due to increased role conflicts and restrictions on personal freedom (48). Shandong Province is a region historically associated with Confucian traditions and conservative family values, which may further amplify the psychological consequences of marital dissatisfaction. Accordingly, the present study aimed to examine this moderated mediation model among Chinese married adults with young children in Shandong Province, a culturally meaningful context for investigating the factors associated with underlying phubbing. Based on our theoretical model and literature review, we hypothesized (see **Figure 1**):

**Figure 1:**
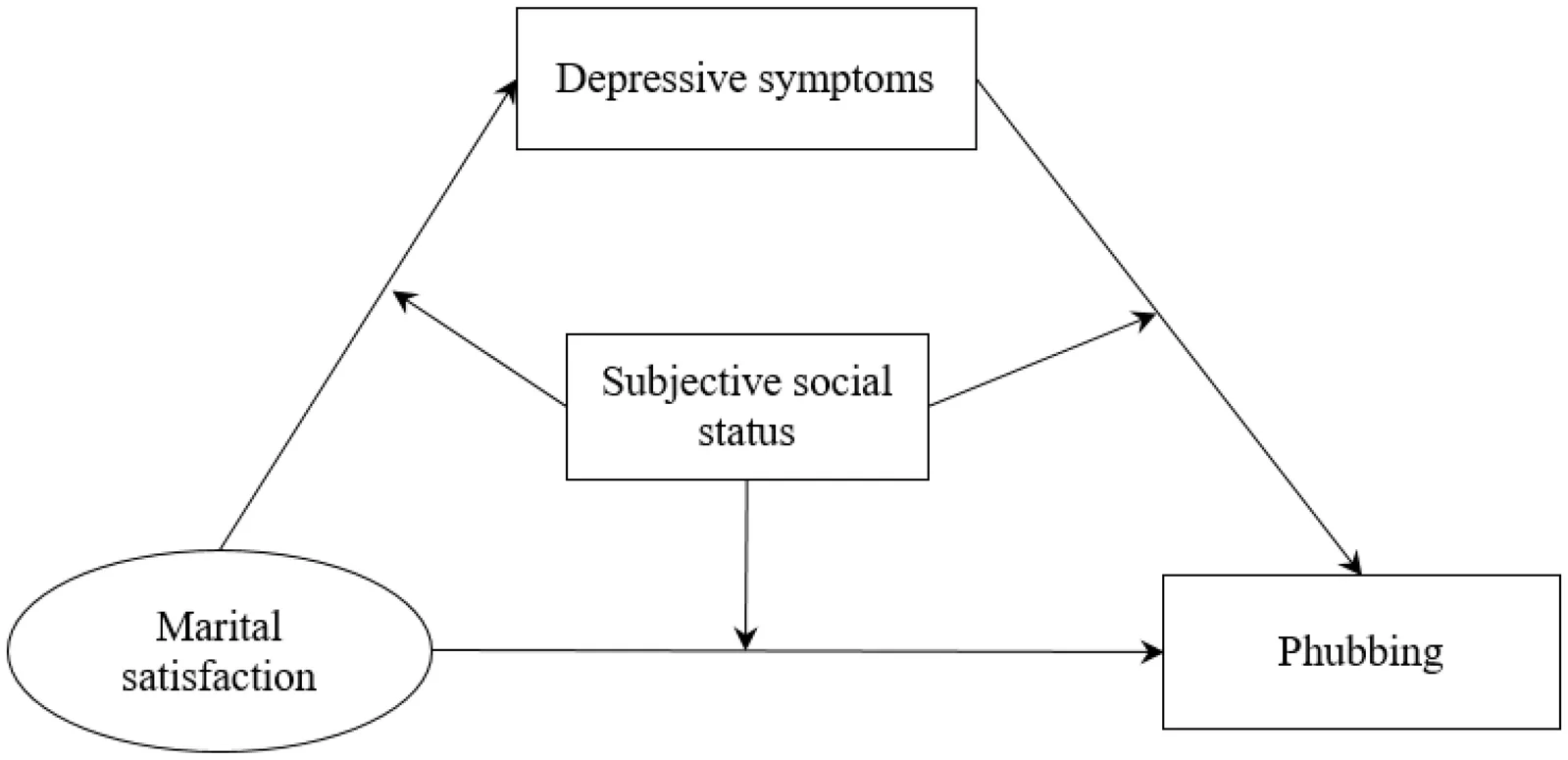
The proposed conceptual moderated mediation model of the present study.

H1: Marital satisfaction would be negatively associated with phubbing among married adults.
H2: Depressive symptoms would mediate the relationship between marital satisfaction and phubbing among married adults.
H3: SSS would moderate the direct association between marital satisfaction and phubbing.
H4: SSS would moderate the indirect association between marital satisfaction and phubbing through depressive symptoms.

## Methods

### Study design and participants

This study adopted a cross-sectional survey design. The target population was married adults aged 18–50 years residing in Shandong Province, China, who had at least one child aged between one and three years. Participants were eligible for inclusion if they met the following criteria: (1) being currently married; (2) being aged between 18 and 50 years; (3) having at least one child aged between one and three years; (4) residing in Shandong Province; and (5) being able to complete the online questionnaire independently. Participants were excluded if they (1) did not meet the above inclusion criteria; (2) failed to complete the questionnaire; or (3) provided invalid responses. A total of 592 questionnaires were initially collected, of which 27 were excluded for completion time under 5 minutes or highly patterned responses, yielding a final analytic sample of 565 (31.85 ± 4.85, range=20–50 years). The sample comprised 185 males (32.7%) and 380 females (67.3%). There were 120 (21.2%) adults from urban areas and 445 (78.8%) from rural areas. Nearly two thirds of the participants had obtained a junior college diploma or university degree (n=371, 65.7%), followed by a senior high school degree (n=107, 18.9%), a master’s degree or above (n=52, 9.2%) and a junior high school degree or below (n=35, 6.2%).

### Measures

#### Marital satisfaction

The marital satisfaction subscale of Olson Marital Quality Questionnaire was used to assess participants’ satisfaction with their marriage (49). This scale includes 10 items (e.g. “I’m very satisfied with the responsibilities the couple takes in the marriage” and “I’m very satisfied with our leisure-time activities and the time we spend together”). Items are rated on a 5-point Likert scale ranging from 1 (strongly disagree) to 5 (strongly agree). Total scores were calculated by summing all the item scores and higher scores indicate greater marital satisfaction. The marital satisfaction subscale demonstrated good reliability in the original validation study (Cronbach’s α = 0.86; 4-week test-retest reliability = 0.86) (49). The scale has also been used in Chinese samples to assess marital satisfaction among Chinese populations (50). In the present study, the Cronbach’s α of this scale was 0.81.

#### Depressive symptoms

Depressive symptoms were assessed using the short Chinese version of Center for Epidemiologic Studies Depression Scale (CES-D) (51), originally developed by Radloff (52). The original 20-item CES-D was designed to measure the frequency of current depressive symptoms in the general population, with demonstrated high internal consistency (Cronbach’s α ≥ 0.80 across all subgroups) and adequate test-retest reliability (r ≥.40), as well as strong concurrent and construct validity. The 9-item Chinese short version of CES-D assesses the frequency of depressive symptoms experienced during the previous week and includes both negatively and positively worded items, such as “I felt depressed” and “I was happy.” It demonstrated satisfactory reliability (Cronbach’s α = 0.85–0.88; test-retest r = 0.49, *p* < 0.001) and structural validity based on a two-factor model (χ²/df = 2.39, CFI = 0.979, RMSEA = 0.053), along with strong convergent validity as indicated by high correlations with the original 20-item scale (r = 0.94–0.96) and adequate criterion validity (r = 0.61 with the Beck Depression Inventory, *p* < 0.001). Participants rated each item on a 4-point Likert scale ranging from 0 (rarely or none of the time; less than 1 day) to 3 (most or all of the time; 5–7 days). Two positively worded items were reverse-coded prior to computing the total score. After reverse scoring, all nine items were summed, with total scores ranging from 0 to 27. Higher scores indicate more severe depressive symptoms. In the present study, the Cronbach’s α of this scale was 0.75.

#### Phubbing

The Chinese version of the Generic Scale of Phubbing (GSP) (53), originally developed by Chotpitayasunondh and Douglas (54), was used to evaluate participants’ phubbing. In line with prior conceptualizations, phubbing is characterized by the tendency to ignore or disengage from others during social interaction by prioritizing smartphone use, which may in turn undermine interpersonal engagement and communication quality. The 15-item GSP scale consists of four dimensions: nomophobia (4 items), interpersonal conflict (4 items), self-isolation (4 items) and problem acknowledgement (3 items). Representative items include: (a) nomophobia, such as “I feel anxious if my phone is not nearby”; (b) interpersonal conflict, such as “I have conflicts with others because I am using my phone”; (c) self-isolation, such as “I would rather pay attention to my phone than talk to others”; and (d) problem acknowledgement, such as “I pay attention to my phone for longer than I intend to do so.” All items were rated on a 5-point Likert scale ranging from 1 (not at all true for me) to 5 (extremely true of me). Total scores ranged from 15 to 75, with higher scores indicating greater severity of phubbing. The original version demonstrated good structural validity (χ²/df = 2.20, CFI = 0.97, GFI = 0.93, RMSEA = 0.06), adequate composite reliability (CR > 0.70) and convergent validity (AVE > 0.50) for all subscales, and strong concurrent validity with a measure of problematic smartphone use (r = 0.85, *p* < 0.001) (54). The Chinese version (53) also showed satisfactory structural validity (χ²/df = 2.59, GFI = 0.93, CFI = 0.93, RMSEA = 0.06), with Cronbach’s α coefficients ranging from 0.71 to 0.84 across subscales and test-retest reliability coefficients ranging from 0.68 to 0.85 (*p* < 0.01). In the present study, the Cronbach’s α of this scale was 0.93.

#### Subjective social status

The MacArthur Scale of Subjective Social Status was used to measure SSS (32). Participants were presented with a picture of a 10-rung ladder with the instructions: “Imagine this ladder below shows how Chinese society is set up. Think of where your family would be on this ladder, with higher rungs indicating higher status (more money, more education, and better jobs). Please select the rung on the ladder where you think your family stands in the social hierarchy (1 = lowest status, 10 = highest status).” Higher scores indicate higher levels of SSS. The MacArthur Scale has demonstrated good construct validity and has been widely used in both Western and Chinese populations to assess perceived social standing (55, 56).

### Procedure

Shandong is a coastal province in East China and one of the country’s most economically developed regions. Smartphone ownership and daily mobile internet use are widespread among residents aged 18-50, particularly among younger adults. In addition, Shandong has well-developed mobile network infrastructure, which provides a suitable context for examining smartphone-related behaviors. Data were collected in September 2021 through Sojump, a widely used online survey platform in China. A convenience sampling approach was adopted, in which eligible participants were recruited via questionnaire links distributed in WeChat groups. They were then invited to complete an online self-report questionnaire. Before completing the questionnaire, participants were informed of the study purpose, the voluntary nature of participation, and the confidentiality of their responses. After providing informed consent, participants completed a self-report questionnaire, which took approximately 15–20 minutes. As all survey items were set as mandatory within the Sojump platform, participants were required to respond to every item before submission. Consequently, no missing data were present in the dataset. The study was approved by the Research Ethics Committee of School of Public Health, Shandong University and was conducted in accordance with the ethical standards of the 1964 Declaration of Helsinki and its later amendments.

### Statistical analyses

Descriptive analysis (mean, standard deviation, skewness and kurtosis) and Pearson’s correlation analyses were conducted for all studied variables. The skewness and kurtosis values indicated all continuous variables were approximately normally distributed (e.g. ∣skewness∣<0.8, ∣kurtosis∣<0.9) (57). The PROCESS macro for SPSS (Model 4) was first applied to examine the mediating role of depressive symptoms in the relationship between marital satisfaction and phubbing (58). Subsequently, PROCESS macro for SPSS (Model 59) was employed to determine whether SSS moderated each paths in the mediation model, with all the continuous variables standardized prior to data analyses. A moderated mediation effect was supported if the index of moderated mediation was significant (i.e., its bootstrap 95% CI did not include zero), indicating that the indirect effect of marital satisfaction on phubbing via depressive symptoms varied significantly as a function of SSS (59). As a robustness check, the moderated mediation model was also re-estimated using Model 15, in which SSS was specified to moderate the *b* path and the direct path but not the *a* path. A bias-corrected bootstrap confidence interval (CI) was generated by the PROCESS macro for SPSS with bootstrapping procedure based on 5,000 resamples from the data. Simple slope analysis was conducted to further illustrate the interaction effect. Low and high levels of SSS were defined as 1 SD below and above the mean, respectively. Statistical analyses were conducted using SPSS 24.0 v (IBM SPSS, IBM Corp, Armonk, NY, USA).

## Results

### Preliminary analyses

Descriptive statistics and bivariate correlation coefficients among all study variables are presented in **Table 1**. Sex was significantly associated with depressive symptoms (*r* = -0.09, *p*<0.05). Age was negatively associated with depressive symptoms (*r* = -0.09, *p*<0.05) and phubbing (*r* = -0.14, *p*<0.01). Marital satisfaction was negatively related to depressive symptoms (*r* = -0.32, *p*<0.01) and phubbing (*r* = -0.31, *p*<0.01), while positively associated with SSS (*r* = 0.11, *p*<0.01). Depressive symptoms were positively correlated with phubbing (*r* = 0.46, *p*<0.01) and negatively related to SSS (*r* = -0.13, *p*<0.01). Finally, phubbing was negatively linked to SSS (*r* = -0.13, *p*<0.01).

**Table 1:** Descriptive statistics and bivariate correlation between variables (n=565).

Variables	1	2	3	4	5	6
1. Sex	1					
2. Age	0.01	1				
3. Marital satisfaction	-0.04	-0.01	1			
4. Depressive symptoms	-0.09^*^	-0.09^*^	-0.32^**^	1		
5. Phubbing	-0.07	-0.14^**^	-0.31^**^	0.46^**^	1	
6. SSS	-0.01	-0.06	0.11^**^	-0.13^**^	-0.13^**^	1
M	–	31.85	34.53	19.33	38.14	5.25
SD	–	4.85	6.67	4.05	12.09	1.92
Skewness	–	0.73	0.47	0.27	0.34	0.12
Kurtosis	–	0.81	0.33	0.41	0.22	0.18

Sex was dummy coded as 1=male and 2= female.

M, mean; SD, standard deviation; SSS, subjective social status.

^**^ *p*<0.01, *p<0.05.

### Testing for the mediation model

The mediating role of depressive symptoms in the association between marital satisfaction and phubbing was examined using Model 4 in the PROCESS macro for SPSS, with 5,000 bootstrap resamples. Sex and age were included as covariates. As shown in **Table 2**, marital satisfaction was negatively and significantly associated with depressive symptoms (path a: β=-0.33, *t* = -8.33, *p* < 0.001), which in turn was related to phubbing (path b: β=0.39, *t* = 9.89, *p* < 0.001). The direct effect of marital satisfaction on phubbing was also significant (path c′: β= -0.18, *t* = -4.75, *p* < 0.001). The indirect effect of marital satisfaction on phubbing via depressive symptoms were -0.13 (95% CI [−0.17, −0.09]), as the confidence interval did not include zero. These findings indicate that depressive symptoms partially mediated the association between marital satisfaction and phubbing.

**Table 2:** Mediation model test for depressive symptoms.

Outcome variables	Predictors	*R^2^*	*F*	β	LLCI	ULCI	*t*
Depressive symptoms	Sex	0.12	26.31^***^	-0.21	-0.38	-0.05	-2.50^*^
	Age			-0.02	-0.03	-0.01	-2.31^*^
	Marital satisfaction			-0.33	-0.41	-0.25	-8.33^***^
Phubbing	Sex	0.25	47.09^***^	-0.10	-0.25	0.06	-1.23
	Age			-0.02	-0.04	-0.01	-2.92^**^
	Marital satisfaction			-0.18	-0.26	-0.11	-4.75^***^
	Depressive symptom			0.39	0.31	0.46	9.89^***^

Sex was dummy coded as 1=male and 2= female.

LLCI, lower limit of the 95% confidence interval; ULCI, upper limit of the 95% confidence interval.

^*^ *p*<0.05; ^**^p<0.01; ^***^p<0.001.

### Testing for moderated mediation model

A moderated mediation model was estimated using Hayes’ PROCESS macro (Model 59) for SPSS. All continuous variables were standardized, and sex and age were included as covariates, with 5,000 bootstrap resamples. As displayed in **Table 3**, the interaction term of marital satisfaction and SSS was not significantly associated with depressive symptoms (β=0.05, *t* = 1.28, *p*>0.05), indicating that SSS did not moderate the path from marital satisfaction to depressive symptoms (path a). However, the interaction term was significantly correlated to phubbing (β=-0.10, *t* = -2.63, *p* < 0.01), indicating that SSS moderated the direct path from marital satisfaction to phubbing (path c′). In addition, the interaction term of depressive symptoms and SSS was significantly associated with phubbing (β=-0.11, *t* = -2.98, *p* < 0.01), indicating that SSS also moderated the path from depressive symptoms to phubbing (path b). As a robustness check, we re-estimated the model using Model 15. The index of moderated mediation remained significant (index = 0.04, BootSE = 0.03, 95% CI [0.004, 0.078]), consistent with the results obtained from Model 59, further supporting the conclusion that the indirect effect of marital satisfaction on phubbing via depressive symptoms varies significantly as a function of SSS. Specifically, the indirect effect was stronger among individuals with lower SSS (effect= -0.16, BootSE = 0.03, 95% CI=[-0.23, -0.11]) than among those with higher SSS (effect= -0.09, BootSE = 0.03, 95% CI=[-0.14, -0.04]).

**Table 3:** Moderated mediation model of marital satisfaction on phubbing.

Predictors	Model 1 (depressive symptom)			Model 2 (phubbing)		
	β	*t*	[LLCI, ULCI]	β	*t*	[LLCI, ULCI]
Sex	-0.22	-2.63^**^	[-0.39, -0.06]	-0.07	-0.96	[-0.23, 0.08]
Age	-0.02	-2.54^*^	[-0.04, -0.01]	-0.02	-2.68^**^	[-0.03, -0.01]
Marital satisfaction	-0.32	-8.03^***^	[-0.40, -0.24]	-0.18	-4.54^***^	[-0.25, -0.10]
SSS	-0.09	-2.35^*^	[-0.17, -0.02]	-0.07	-1.85	[-0.14, 0.01]
Marital satisfaction×SSS	0.05	1.28	[-0.03, 0.12]	-0.10	-2.63^**^	[-0.17, -0.02]
Depressive symptoms				0.38	9.74^***^	[0.30, 0.46]
Depressive symptoms ×SSS				-0.11	-2.98^**^	[-0.18, -0.03]
*R^2^*	0.14			0.27		
*F*	17.50^***^			29.52^***^		
Conditional direct effect analysis at values of the moderator	SSS values		Effect	*SE*	LLCI	ULCI
	-1 (*M-*1 *SD*)		-0.06	0.06	-0.18	0.06
	0 (M)		-0.16	0.04	-0.24	-0.09
	1 (*M+*1 *SD*)		-0.26	0.05	-0.36	-0.16
Conditional indirect effect analysis at values of the moderator	SSS values		Effect	*BootSE*	BootLLCI	BootULCI
	-1 (*M-*1 *SD*)		-0.19	0.04	-0.27	-0.13
	0 (M)		-0.13	0.02	-0.17	-0.08
	1 (*M+*1 *SD*)		-0.08	0.03	-0.13	-0.03

SSS, subjective social status; *SE*, standard error; *SD*, standard deviation; LLCI, lower limit of the 95% confidence interval; ULCI, upper limit of the 95% confidence.

^*^ *p* < 0.05, ^**^p < 0.01, ^***^p < 0.001.

To facilitate the interpretation of the moderating effect of SSS, the relationship between marital satisfaction and phubbing, and the association of depressive symptoms with phubbing were plotted separately at high (M + 1SD) and low (M − 1SD) levels of SSS (**Figure 2**). Simple slopes analyses indicated that among individuals with high SSS, marital satisfaction was significantly and negatively associated with phubbing (β*_simple_* = -0.42, *t* = -4.16, *p* < 0.001). In contrast, this association was not significant among those with low SSS (β*_simple_* = 0.04, *t* = 0.46, *p* = 0.65). Regarding the second path, depressive symptoms were positively associated with phubbing among individuals with low SSS (β*_simple_* = 0.62, *t* = 6.99, *p* < 0.001), whereas this association was not significant among those with high SSS (β*_simple_* = 0.11, *t* = 1.13, *p* = 0.26).

**Figure 2:**
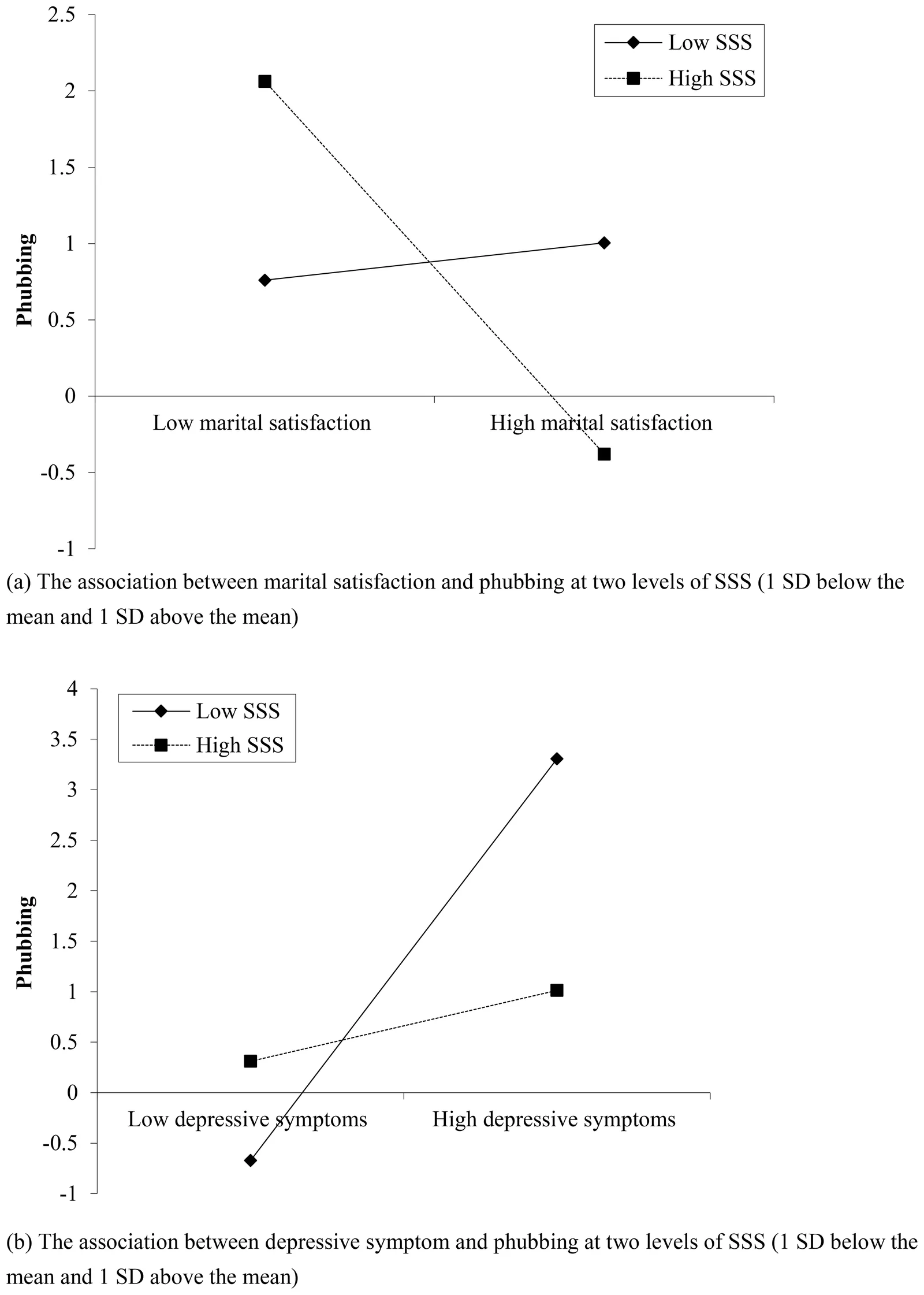
Plot of the link marital satisfaction and phubbing via depressive symptoms at two levels of SSS. **(a)** The association between marital satisfaction and phubbing at two levels of SSS (1 SD below the mean and 1 SD above the mean). **(b)** The association between depressive symptom and phubbing at two levels of SSS (1 SD below the mean and 1 SD above the mean).

## Discussion

Based on the existing theories, the current study examined the indirect association between marital satisfaction and phubbing via depressive symptoms among Chinese married adults, as well as the moderating role of SSS in the indirect association. Low marital satisfaction was associated with poorer adjustment, including elevated depressive symptoms and greater phubbing. Moreover, depressive symptoms mediated the association between marital satisfaction and phubbing, and SSS moderated this mediation model. Specifically, SSS moderated the direct path (from marital satisfaction to phubbing) and the second path (from depressive symptoms to phubbing) of the mediation model. Findings demonstrate variability in the associations of marital satisfaction with depressive symptoms and phubbing as a function of SSS in Chinese married adults.

The results showed that marital satisfaction was significantly and negatively associated with phubbing in married adults, which supported Hypothesis 1. This finding is consistent with prior research indicating that decreased relationship quality was a risk factor for phubbing (60). From the perspective of interpersonal theory, unmet needs within the marital relationship may motivate individuals to use the Internet as a substitute for real-world couple contact, or as a means of escape when they feel dissatisfied with their marital relationship (14). Married adults who reported lower marital satisfaction or who evaluated their relationship with their partners negatively may experience greater conflict and less closeness, resulting in unmet relationship needs. Individuals who perceive lower relationship quality may become less responsive to their spouses and more absorbed in mobile phone use (60).

Consistent with Hypothesis 2, depressive symptoms were found to mediate the association between marital satisfaction and phubbing. Specifically, lower marital satisfaction was associated with higher levels of depressive symptoms, which in turn were associated with greater phubbing. These findings suggest depressive symptoms as one factor associated with the tendency for married adults with lower marital satisfaction to engage in higher levels of phubbing. Drawing on the coping-oriented drinking process described in the alcohol-marriage literature, in which marital distress prompts affect-regulation attempts such as drinking to cope (61), individuals under marital stress may similarly turn to using mobile phones to regulate negative emotions and seek emotional relief for unmet relational needs. This pattern is broadly consistent with previous findings suggesting that problematic Internet use is associated with psychological distress stemming from unmet relational needs (62).

Regarding the individual paths within the mediation model, the results revealed that higher marital satisfaction was associated with fewer depressive symptoms. This finding is in accordance with prior research (22–26) and is consistent with the MDMD (27). According to this model, marital satisfaction may serve as a protective factor against depressive symptoms, whereas marital dissatisfaction may function as a precipitating factor. The MDMD proposes two etiological pathways through which a distressed marriage may be associated with depressive symptoms (63, 64). First, adults with lower marital satisfaction may be more likely to experience hostility, which is associated with an elevated risk of depressive symptoms. Second, spouses in distressed relationships tend to provide and receive less social support, placing them in a more vulnerable position with respect to depressive symptoms.

In addition, the results indicated that higher levels of depressive symptoms were associated with greater phubbing, which is consistent with the previous finding (65) and with the cognitive-behavioral model of pathological Internet use (31). Individuals with more pronounced depressive symptoms were inclined to exhibit problematic smartphone use behaviors and to engage in phubbing more frequently (66, 67). Given that online communication may feel less threatening than face-to-face interaction, individuals with elevated depressive symptoms may prefer digital social contact over in-person communication (68). From the perspective of the self-medication model, married adults with high levels of depressive symptoms may use mobile phones, viewed here as an emotion-regulation strategy rather than an addictive behavior, as a more accessible and less harmful coping strategy compared to other means (69).

Consistent with Hypothesis 3, SSS moderated the direct association between marital satisfaction and phubbing. A previous study found that perceived SES moderated the association between marital quality and life satisfaction among Chinese married residents (36). In the present study, the negative association between marital satisfaction and phubbing was significant only among individuals with higher SSS, not among those with lower SSS. This pattern does not support the cumulative risk model. Rather, it suggests that married adults with higher SSS may benefit more from high marital quality, such that greater satisfaction is associated with less phubbing. The rewards of a satisfied marriage were greater for individuals who had a high level of SSS, while disadvantages of a bad-quality marriage did not lead to phubbing for persons with lower SSS. One possible explanation is that individuals with higher SSS have access to greater social resources and support derived from their marriage, which may buffer against phubbing. For those with lower SSS, however, the positive role of marital satisfaction may not be sufficient to offset the constraints associated with socioeconomic disadvantage, and marital satisfaction may thus show no significant association with phubbing in this group. It is also noteworthy that SSS did not moderate the first path of the mediation model (from marital satisfaction to depressive symptoms), suggesting that the protective role of marital satisfaction against depressive symptoms did not differ significantly by SSS level.

Consistent with Hypothesis 4, SSS moderated the indirect association between marital satisfaction and phubbing via depressive symptoms. Specifically, the association between depressive symptoms and phubbing was significant only among individuals with lower SSS. The finding suggests that lower SSS may exacerbate the association between depressive symptoms and phubbing. Individuals with lower SSS who reported greater depressive symptoms also exhibited more phubbing. This is consistent with prior research showing that family SES moderated the relationship between social anxiety and problematic online gaming among Chinese undergraduates (70). This research also indicated that the indirect effect of neuroticism on problematic online gaming via social anxiety was only significant for those with lower family SES. Individuals with lower SSS may have more limited access to social capital or resources when experiencing depressive symptoms, making them more likely to regulate negative affect through excessive mobile phone use and to phub others during face-to-face interactions (69).

### Study implications

The present study offers implications for prevention and intervention programs targeting phubbing among Chinese married adults. From a research perspective, this study extends the existing literature by elucidating the indirect and conditional associations linking marital satisfaction to phubbing, identifying depressive symptoms as a mediating pathway and SSS as a boundary condition. The moderated mediation framework applied here provides a more nuanced understanding of phubbing than prior work focusing predominantly on the phubee perspective, by elucidating the psychological and social factors associated with phubbing among married adults. From a clinical perspective, the findings suggest that interventions targeting marital dissatisfaction and depressive symptoms may represent meaningful targets for attention. Clinicians working with couples, particularly those with young children, should consider addressing both relationship quality and emotional well-being as part of treatment. Individuals with lower SSS may benefit from additional support, as the indirect association between marital dissatisfaction and phubbing through depressive symptoms appeared comparatively stronger in this group. From a social perspective, the results underscore the relevance of socioeconomic inequality in shaping technology-related interpersonal behaviors. Policies and community programs aimed at reducing social disparities and supporting disadvantaged families may contribute to healthier marital and digital behaviors at the population level.

### Limitations and recommendations

Despite these practical implications, several limitations should be acknowledged. First, the cross-sectional design precludes causal inferences regarding the hypothesized moderated mediation model. For example, although the theoretical and empirical literature supports treating marital satisfaction as an antecedent of phubbing, the cross-sectional nature of this study precludes definitive causal conclusions. Future research employing longitudinal designs (e.g., multi-wave assessments over 6–12 months), laboratory-based experimental manipulation of marital stress through couple discussion tasks, or experience-sampling methods (ESM) conducted over 2–4 weeks would permit stronger causal inference (71). Second, all variables were assessed via self-report, which may introduce recall bias and social desirability bias (72). Future studies should incorporate multiple informants (73) or objective measures (e.g., smartphone usage logs) to triangulate findings. Third, there are other potential factors involved in the path between marital satisfaction and phubbing, such as attachment styles or marital conflict frequency (74). Future research incorporating these additional factors would strengthen understanding of the correlates of phubbing in married couples.

## Conclusions

In summary, this study indicated that marital satisfaction was negatively associated with phubbing among married adults. The current study further advances understanding of the factors and boundary conditions underlying this association. Specifically, higher marital satisfaction was associated with fewer depressive symptoms, which in turn were associated with less phubbing. SSS moderated both the direct association between marital satisfaction and phubbing and the association between depressive symptoms and phubbing. These findings contribute to guiding prevention and intervention efforts targeting phubbing in Chinese married adults.

## Data Availability

The raw data supporting the conclusions of this article will be made available by the authors, without undue reservation.
